# Late Pleistocene-Holocene vegetation history and anthropogenic activities deduced from pollen spectra and archaeological data at Guxu Lake, eastern China

**DOI:** 10.1038/s41598-020-65834-z

**Published:** 2020-06-09

**Authors:** Zhenwei Qiu, Hongen Jiang, Lanlan Ding, Xue Shang

**Affiliations:** 1 National Museum of China, Beijing, 100006 China; 2 Department of Archaeology and Anthropology, University of Chinese Academy of Sciences, Beijing, 100049 China; 3 Helu Relics Museum, Wuxi, 214161 China

**Keywords:** Palaeoclimate, Climate-change adaptation, Palaeoecology, Plant domestication

## Abstract

This study presents high-resolution pollen and charcoal records from Guxu Lake in the Taihu Lake Basin, eastern China, spanning the last 23,000 years. The sedimentary sequences revealed dynamic terrestrial and lacustrine environments during 23.0-11.7 cal ka BP, the climate was relatively cold and dry, and the vegetation was dominated by evergreen-deciduous broadleaf and coniferous mixed forest. During 11.7-4.4 cal ka BP, the *Quercus-* and *Castanopsis*-dominated evergreen-deciduous broadleaf mixed forest expanded, while the Poaceae and *Artemisia* were still the major terrestrial herbs under warmer and more humid conditions. After this period, the climate became relatively cool and dry again, and the vegetation landscape was comparatively stable, as it remains today. Wild rice likely grew before Neolithic humans occupied this area. The variations in *Oryza*-type Poaceae pollen spectra and distributions of Neolithic archaeological sites indicate rice agriculture may have first appeared and developed with human occupation in ca. 7.0-4.4 ka BP. During the historical period, beginning approximately 4 ka BP, a clear signal of intensified anthropogenic disturbance is evident from the clearing of forests, high charcoal concentrations and the presence of rice pollen in large quantities. These results suggest more intensified rice farming was widespread, with increasing human impact on the environment.

## Introduction

Reconstructing the history of vegetation helps shed light on environmental shifts and human adaptations in response to climatic changes, especially during the Last Glacial Maximum and the Holocene. Such reconstruction can also elucidate the response of humans to global changes and suggest ways to coordinate the relationship between man and nature in the future^1^. To date, millennial and orbital-scale events^2,3^, autoecological processes and/or high regional topographical variability^4^ have been considered as the main factors controlling the vegetation changes through hemispherical or semi-hemispherical records from ice cores and speleothems^5,6^, regional and/or local palynological data^7,8^ and microcharcoal^9,10^ and diatom analyses^11,12^. Organic materials (fossil pollen in particular) are often sufficiently well preserved in waterlogged deposits^13^. Lake sediments in particular are considered the primary archives^14^ of proxy data of past environmental change within and around a lake^15^. In addition, these materials are often the richest source of information on the subsistence strategies of prehistoric inhabitants^16,17^.

**Figure 1:**
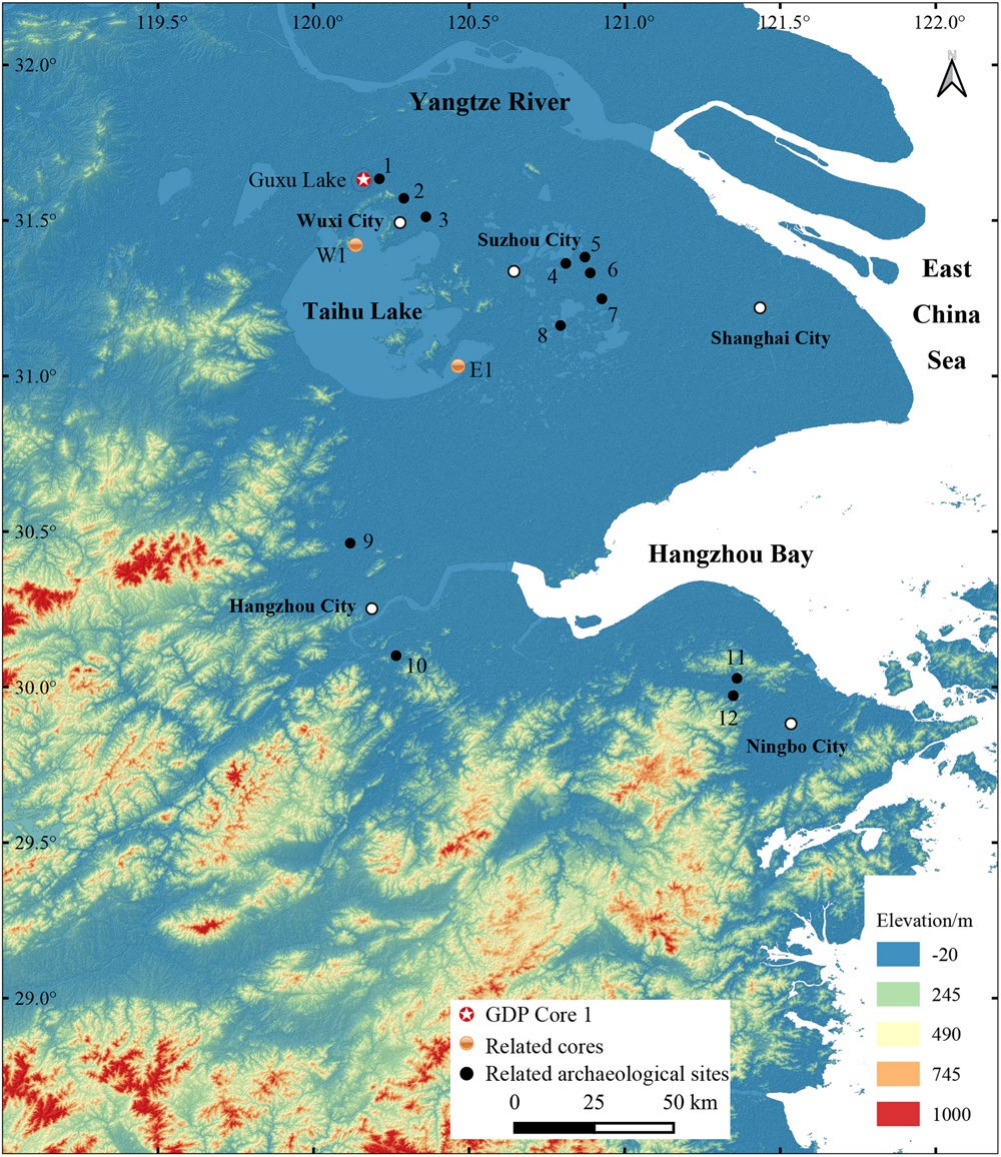
Locations of the GDP Core 1 and related cores and archaeological sites. 1. Yangjia site; 2. Chidun site; 3. Pengzudun site; 4. Caoxieshan site; 5. Chuodun site; 6. Zhumucun site; 7. Jiangli site; 8. Chenghu site; 9. Maoshan site; 10. Kuahuqiao site; 11. Tianluoshan site; 12. Hemudu site. The map was generated using QGIS v3.12 (https://qgis.org/en/site/) and Adobe Illustrate CC 2019 (https://www.adobe.com/cn/products/illustrator.html). The SRTM 90 m DEM data set is provided by Geospatial Data Cloud site, Computer Network Information Center, Chinese Academy of Sciences (http://www.gscloud.cn).

The Taihu Lake Basin, located in the lower reaches of the Yangtze River, East China, serves as an example. Many studies on paleoclimatic and paleoenvironmental changes of the Taihu Lake Basin have been carried out through the analysis of sedimentary cores from the eastern, southern and western parts of the area (Fig. 1). In these studies, multi-proxy records of pollen, diatom, carbonate, TOC, TN and δ^13^C of organic matter, grain size and magnetic susceptibility analyses were applied^e.g.^^18–20^. However, most of these studies focused on the history of the climate and vegetation during the Holocene^e.g.^^21–24^, whereas there are remarkably few continuous lake sediment records that extend through to the Last Glacial Maximum in this area to date.

Moreover, Neolithic archaeological research has suggested that ancient people occupied this area through the Majiabang (7.0-5.8 ka BP)^25^, Songze (5.8-5.3 ka BP)^26^, Liangzhu (5.3-4.3 ka BP)^27^ and even Guangfulin-Qianshanyang (4.3-4.0 ka BP)^28^ periods. These cultures were based on rice farming^29–32^, which not only changed the prehistoric vegetation landscape in the middle and lower reaches of the Yangtze River but also greatly promoted the development and changes of regional archeological cultures^33,34^. However, studies in this area have focused on the reconstruction of the vegetation history and environmental conditions through natural depositions^12,35^ and deposits in archaeological sites^36–38^, respectively, and few comparative studies of these two subjects have been reported^39^. Thus, the relationships between human activities (especially rice farming in this study) and vegetation history, as well as environmental change, remain unclear. Nevertheless, agriculture is a typical example of human adaptation to the natural environment and human impact on natural vegetation.

Prehistoric cultures and the activities of humans, who continuously occupied the Taihu Lake Basin, were mainly concentrated in the late Neolithic period (approximately 7.0-4.0 ka BP), which has been attributed to the relatively closed geographical environment in the Taihu Lake Basin creating an isolated habitat. Hence, the Taihu Lake Basin, called the “East Asian half-arc for rice agriculture”^40^, serves as an ideal area for the study of regional responses and human adaptations to global change, especially during the Holocene. This Basin also provides an excellent natural base to explore the occurrence and development of rice farming in China and East Asia as well as its relationship to the development of prehistoric culture and environmental changes.

Consider these, we conducted vegetation history and paleoenvironment research work via the Guxu Lake Drilling Project (GDP) in the north of the Taihu Lake Basin, where previous work was scarce. Here, we present the first continuous high-resolution pollen and charcoal records from the GDP Core 1 since the last glacial period (23.0 cal ka BP to present) and reveal the regional vegetation history and the characteristics of environmental evolution, as well as human adaptations, represented by rice agriculture and Neolithic cultures in the northern Taihu Lake Basin. On this basis, the regional response to hemispheric-scale or semi-hemispheric-scale climatic changes was investigated by comparing the data to δ^18^O records from the Greenland ice sheet and East Asian stalagmites.

### Regional settings and site description

Guxu Lake (31°30′55.34″N, 120°07′07.54″E), located in the northern Taihu Lake Basin, is close to the north shore of Taihu Lake, China’s third largest freshwater lake, and lies between the ancient city of Helu and Xushan Hill, in Jiangsu Province, eastern China (Fig. 1). This lake reportedly served as a naval base for training and docking ships of the Wu State in the Spring and Autumn Period (770-476 BC). Today, a water garden project is being developed in the center of Guxu Lake, and the edges of the entire lake is used as a landfill. This area presently experiences a moderate and moist East Asian subtropical monsoon-type climate, with four distinct seasons. The mean annual temperature is 15–17 °C, the annual precipitation is 1000–1400 mm, and the frost-free period lasts for 220–246 days^41^. Today, this area is mostly occupied by cultivated vegetation, rice (*Oryza sativa*) in particular. Northern subtropical mixed evergreen and deciduous broadleaf secondary or successional forests (*Castanopsis*, *Quercus*, *Betula*, and *Liquidambar* are the most representative and dominant species) are present on isolated hills on the Yangtze River Delta plain and on mountains flanking the south and east of this area^42^.

The available paleoenvironmental evidence and pollen records indicate a relatively stable sedimentary context and continuous accumulations that were sensitive to changes in sea level during the generally warm and wet Holocene^e.g.^^43–47^. Moreover, studies have shown that in addition to ancient river deposits, the Holocene sediments in the Taihu Lake Basin were mostly formed after ca. 8.0 ka BP^20^.

## Results

### Chronology

**Figure 2:**
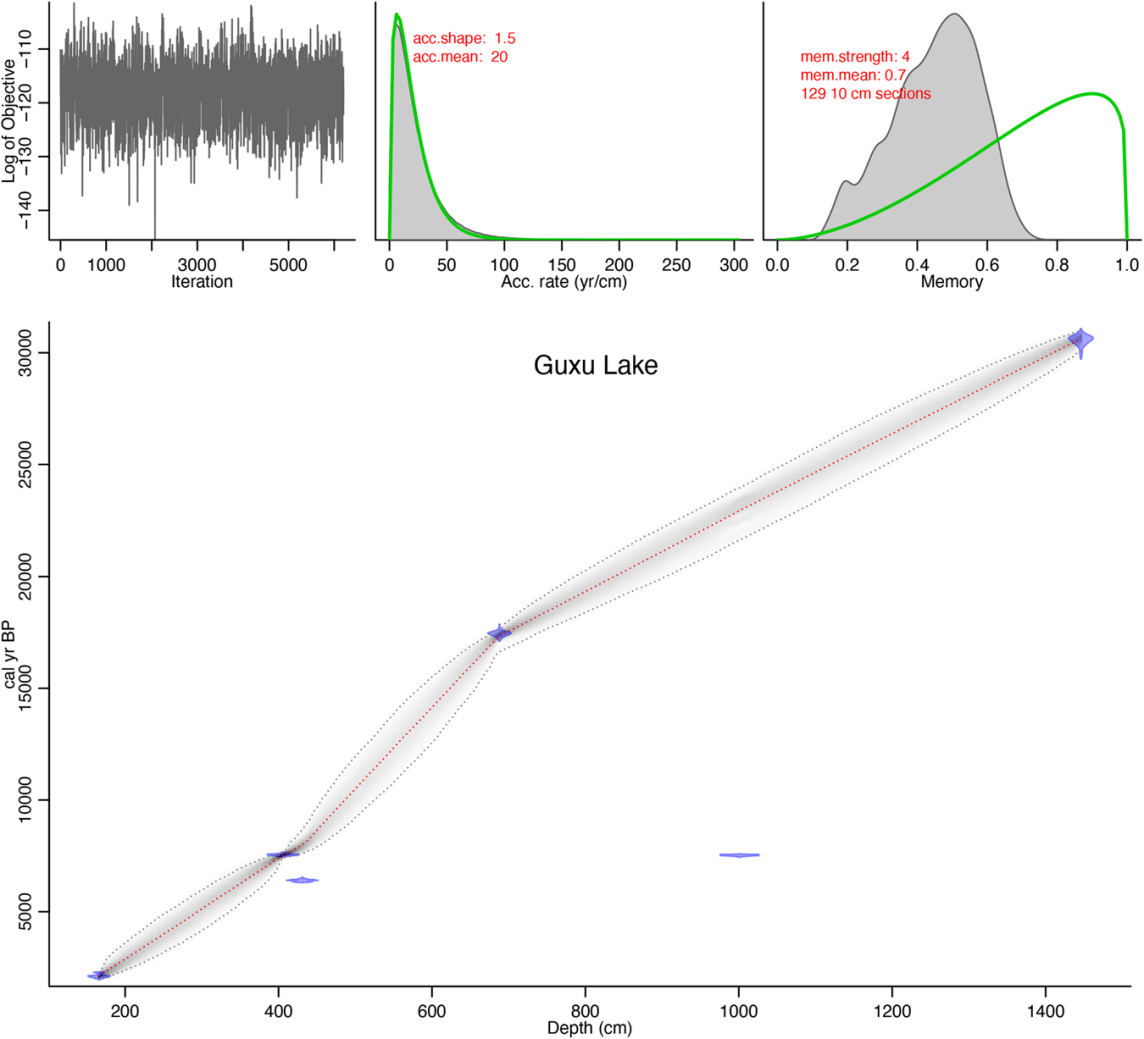
Age-depth model for sedimentary Core 1 of the Guxu Lake obtained by BACON program^48^. Upper panels show the MCMC random walks (left panel, greyscale shading), the prior (green curves) and posterior (grey histograms) distributions for the accumulation rate (middle panel) and memory (right panel). Bottom panel depicts the calibrated 14 C dates (1σ age probability distributions represented in transparent blue) and the age-depth model (darker greys indicate more likely calendar ages; dotted grey lines show 95% confidence intervals; dotted red lines show single ‘best’ model based on the mean age for each depth). The parameter settings are shown at the upper panels (red font).

The AMS dating result (Table 1) was obtained based on TOC extracted from the sediment of the Guxu Lake core. An age-depth model for the 1446 cm of this new Guxu Lake record was built using the Bayesian age-depth modelling program BACON^48^ version 2.3.9.1 and suggests that this record covers at least the last ca. 30 cal ka BP (Fig. 2). Two anomalous dates from 430–432 cm and 1000–1002 cm depth were excluded from the age-depth model because they may have contained organic matter that was not representative of the stratigraphy. Previous studies^49–51^ have suggested that due to sediment storage and reworking before final deposition in deltaic systems in particular, the presence of problematic ^14^C dating is not uncommon.

**Table 1:** AMS ^14^C dating results of sedimentary Core 1 in Guxu Lake.

Lab No.	Depth (cm)	Sample	14 C years(a BP) (T_1/2_ = 5568)	Dendrocalibrated Age (a BP) Ranges (±1δ, 68.2%)	Dendrocalibrated Age (a BP) Ranges (±2δ, 95.4%)
BETA347781	165–167	Organic sediment	2130 ± 30	2152 (68.2%) 2056	2299 (11.1%) 2255
					2159 (84.3%) 2001
BETA348677	405–407	Organic sediment	6680 ± 40	7586 (31.8%) 7556	7615 (95.4%) 7475
				7544 (36.4%) 7509	
BA130926	430–432	Organic sediment	5625 ± 30	6445 (47.9%) 6395	6471 (95.4%) 6315
				6369 (15.8%) 6348	
				6331 (4.5%) 6323	
BA130928	687–689	Organic sediment	14320 ± 40	17546 (68.2%) 17373	17616 (95.4%) 17260
BA140175	1000–1002	Organic sediment	6640 ± 20	7570 (68.2%) 7505	7575 (95.4%) 7480
BETA347782	1445–1447	Organic sediment	26270 ± 130	31070 (68.2%) 30940	31120 (95.4%) 30830

### Pollen and charcoal records

#### Pollen data description

**Figure 3:**
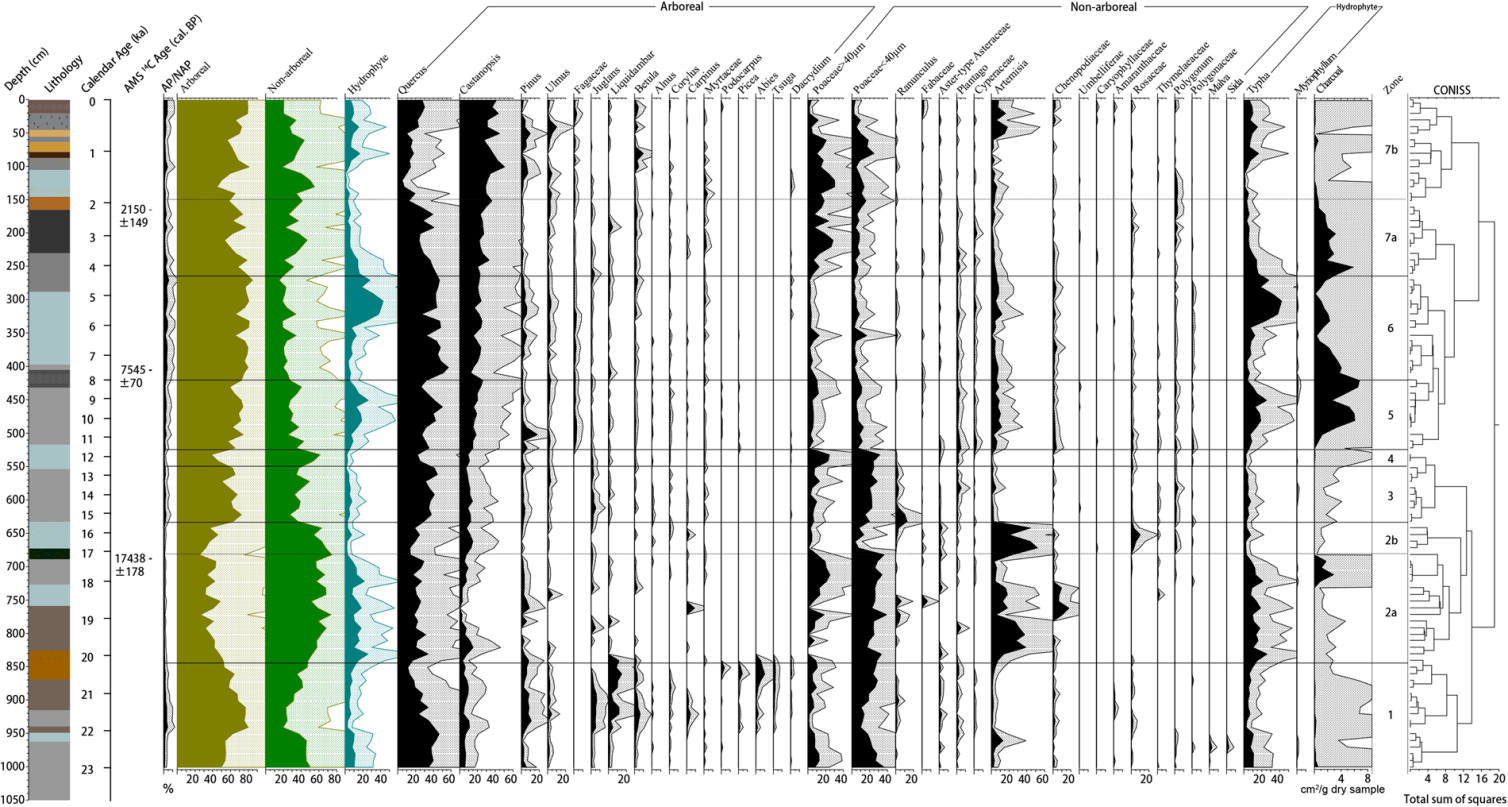
Percentages of palynographs and concentrations of charcoal extracted from GDP Core 1.

**Figure 4:**
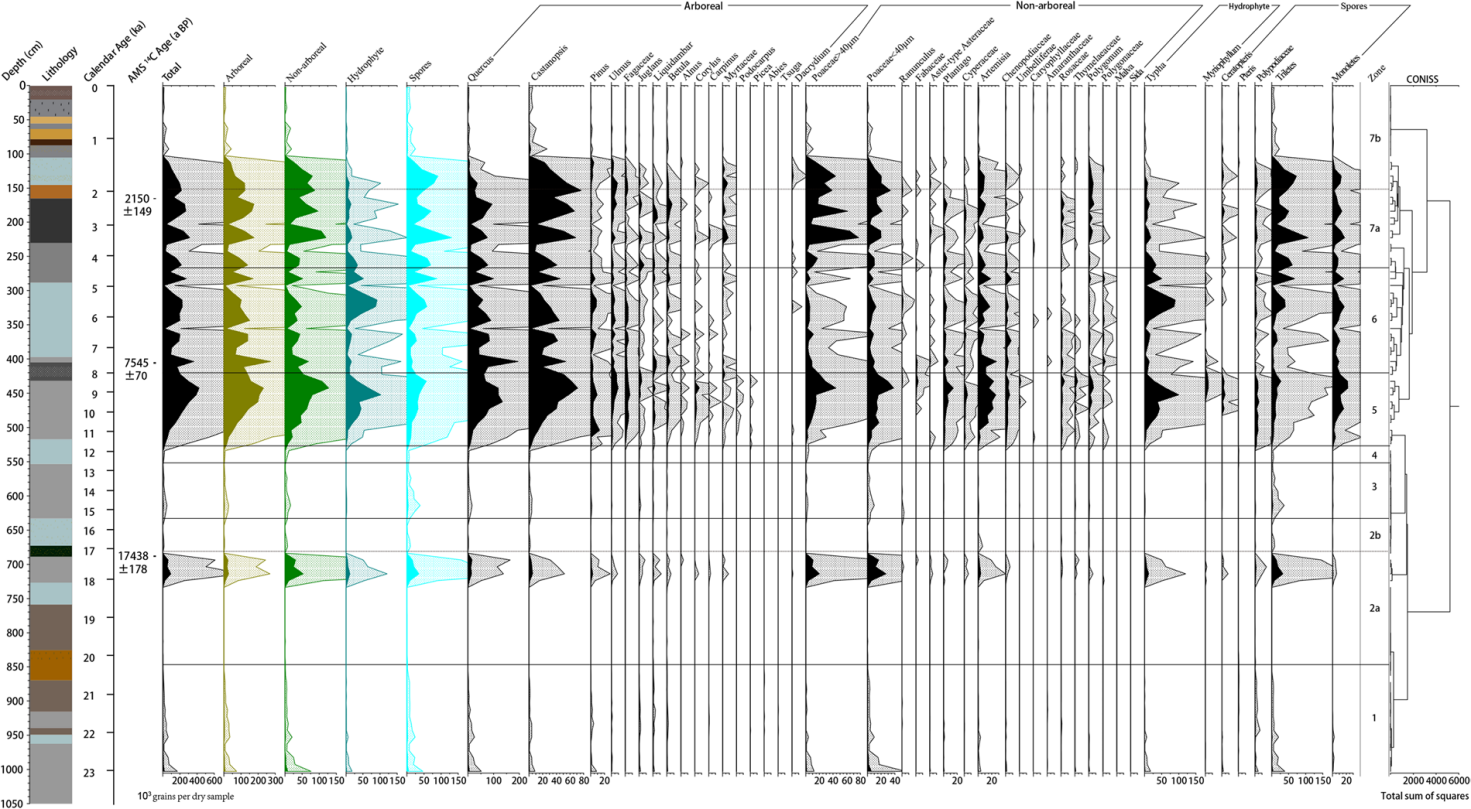
Concentrations of palynographs extracted from GDP Core 1.

Results of fossil sample analysis (101 samples, 67,165 total grains) are shown in the pollen spectra in Figs. 3 and 4. According to the changes in pollen percentages and concentrations, we divided the pollen diagram of the core from Guxu Lake into seven pollen zones, main characteristics of the single pollen zones are presented in Table 2.

**Table 2:** Pollen zone number, depth, age, characteristic pollen assemblages from GDP Core 1.

Pollen zone		Depth (cm)	Age (cal ka BP)	Characteristic pollen assemblages
1		1001–845	23.0-20.0	*Quercus-Castanopsis-Pinus-*Poaceae*-Typha*
2	2a	845–681	20.0-17.2	*Quercus-Castanopsis-Pinus*-Poaceae*-Artemisia-*Chenopodiaceae*-Typha*
	2b	681–635	17.2-15.6	*Quercus-Castanopsis-*Poaceae*-Artemisia*
3		635–550	15.6-12.6	*Quercus-Castanopsis-*Poaceae*- Ranunculus*
4		550–525	12.6-11.7	*Quercus-Castanopsis-*Poaceae
5		525–420	11.7-7.9	*Quercus-Castanopsis-*Poaceae*-Artemisia-Typha*
6		420–265	7.9-4.4	*Quercus-Castanopsis-*Poaceae*-Artemisia-Typha*
7	7a	265–150	4.4-1.9	*Quercus-Castanopsis-*Poaceae
	7b	150–0	1.9-0	*Quercus-Castanopsis-*Poaceae*-Artemisia*

The average (n = 101) total pollen concentration was 76,684 grains per gram dry sample, among which the arboreal pollen concentration was the highest (45,580 grains per gram dry sample), that of terrestrial herbs and shrubs was 21,232 grains per gram dry sample, and that of aquatic herbs was as low as 9872 grains per gram dry sample. Moreover, the total concentration of fern spores was relatively high at 16,556 grains per gram dry sample on average. Generally, pollen assemblages were dominated by arboreal pollen (approximately 61%), whereas the average percentage of terrestrial herb and shrub pollen was close to 39% of the pollen assemblages. Moreover, the average percentage of aquatic pollen, calculated by the value of aquatic pollen to total pollen, was as much as 10%. The concentration of charcoal ranged from 0.003 to 6.757 cm^2^ per gram dry sample (1.109 cm^2^ per gram dry sample on average).

#### PCA of pollen

**Figure 5:**
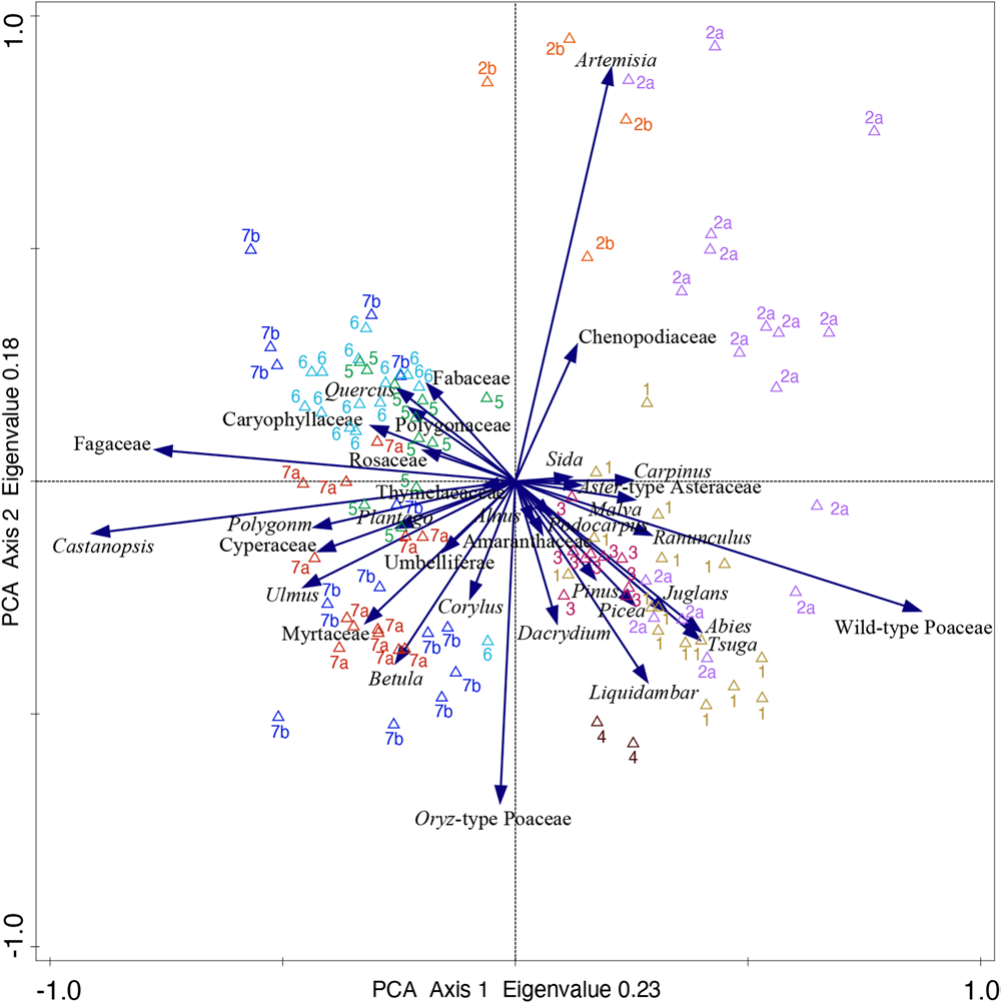
Results of the first two axis of the principal component analysis. Numbers and colors indicate samples of different pollen zones.

The PCA results of pollen taxa and 101 samples of Guxu Lake GDP Core 1 are represented in the pollen diagram and reveal which taxa react similarly to environmental changes (Fig. 5). Together, the first two principal components explained 40.73% of the variation in the pollen data (axis 1: 23.08%; axis 2: 17.65%). Terrestrial herbs and shrubs, as well as coniferous forest, were distributed on the positive end of the first axis while the broadleaved forest mostly scattered on the negative end, which indicates that positive and negative scores based on the Axis 1 (PCA Factor 1) represent dry-cold and humid-warm conditions, respectively. However, no apparent environmental characteristics were revealed by the second axis. Besides, *Oryza*-type Poaceae and *Artemisia* reached the highest scores on the negative and positive ends of Axis 2 (PCA Factor 2) respectively, which probably suggest Man-made and non-human environment, correspondingly.

### Presence of rice pollen

#### Before 11.7 cal ka BP, prior to the Holocene

The percentages of *Oryza*-type Poaceae were rather high, ranging from 0% to 24.9% (approximately 8.6% on average), which is approximately two-fifths that of wild grass Poaceae. In addition, the concentrations of *Oryza*-type Poaceae were notably as low as 1,049 grains per gram dry sample on average (varying from 0 to 20,070 grains per gram dry sample).

#### 11.7–7.9 cal ka BP, early Holocene

The percentages of *Oryza*-type Poaceae were relatively low, varying from 1.5% to 12.3% (approximately 6.7% on average), whereas that of wild grass Poaceae ranged from 5.0% to 13.2% (approximately 8.7% on average). The concentrations of *Oryza*-type Poaceae were notably high, at an average value of 14,543 grains per gram dry sample (varying from 383 to 43,980 grains per gram dry sample), whereas that of wild grass was rather high as well, at an average value of 15,417 grains per gram dry sample (varying from 1,804 to 38,405 grains per gram dry sample).

#### 7.9–4.4 cal ka BP, mid-Holocene

The percentages of *Oryza*-type and wild grass Poaceae decreased to an average value of approximately 4.3% (varying from 2.2% to 12.8%) and approximately 5.3% (ranging from 2.6% to 16.8%), respectively, and their concentrations also decreased to 5,068 grains per gram dry sample on average (varying from 307 to 11,488 grains per gram dry sample) and 6,089 grains per gram dry sample on average (varying from 282 to 12,265 grains per gram dry sample), respectively.

#### After 4.4 cal ka BP, late Holocene

Both the percentages and concentrations of *Oryza*-type Poaceae increased significantly to approximately 14.5% and 16,706 grains per gram dry sample on average (ranging from 1.6% to 30.2% and 4 to 77,518 grains per gram dry sample, respectively). Otherwise, the concentration of charcoal was still rather high, ranging from 0.035 to 5.856 cm^2^ per gram dry sample (approximately 1.141 cm^2^ per gram dry sample on average).

## Discussion

### Vegetation and environmental history in the Guxu Lake area

**Figure 6:**
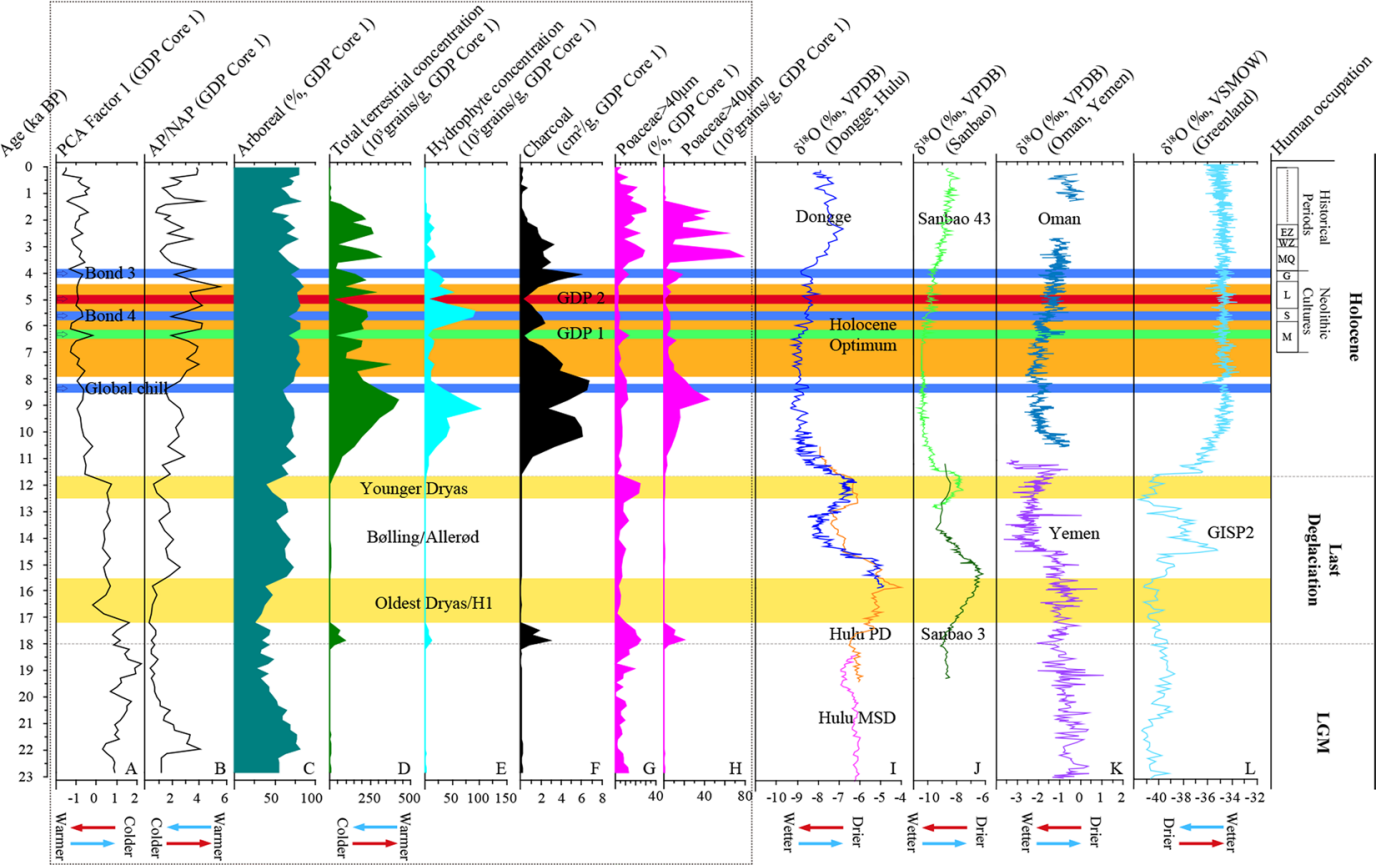
Comparison of deglacial pollen and charcoal records from GDP Core 1 (in the dashed black box, **A–H**) with climate proxy δ^18^O records (**I–L**) from the GISP2 ice core (Greenland) and stalagmites of Northern Hemisphere. The yellow-shaded bars show the OD/H1 and YD cold periods, between which is the BA warm period; the brownish yellow-shaded bar shows the timing of the Holocene Optimum; the green-shaded bar (arrow) “GDP 1” indicates cool event coincident with Neolithic human disturbance, the red-shaded bar (arrow) “GDP 2” suggests probably intensified human activities and the light blue-shaded bars (arrow) show cool oscillations correlated to “Bond events 3 & 4” and “Global chill”. Human occupation consists of two main stages, the Neolithic Cultures and historical periods. M, Majiabang (7.0-5.8 ka BP); S, Songze (5.8-5.3 ka BP); L, Liangzhu (5.3-4.3 ka BP); G, Guangfulin-Qianshanyang (4.3-3.9 ka BP); MQ, Maqiao (ca. 3.9-3.0 ka BP), equivalent to the Xia and Shang Dynasties; WZ, West Zhou Dynasty (ca. 3.0-2.7 ka BP), here is the pre-Wu Culture; EZ, East Zhou Dynasty (ca. 2.7-2.2 ka BP), i.e. the Spring and Autumn and the Warring States Period, here is mainly the Wu, Yue and Chu Cultures.

The palynological data from GDP Core 1 have a broad similarity to the δ^18^O records of a Greenland ice core-Greenland Ice Sheet Project 2 (GISP2)^52^ and stalagmites from Donge Cave^6,53^, Hulu Cave^5^ and Sanbao Cave^3,54^ in eastern and central China; Qunf Cave in southern Oman^55,56^; and Moomi Cave on Socotra Island, Yemen^57^ (Fig. 6). Based on variations in the pollen record and in the charcoal concentration, in conjunction with changes in sedimentary lithology, the evolution of vegetation and paleoclimate changes around Guxu Lake since the Late Pleistocene can be classified into four main stages as follows:

#### 23.0-18 cal ka BP, the Late LGM period

The evergreen-deciduous broadleaf and coniferous mixed forest represented by *Quercus*, *Castanopsis*, and *Pinus* was distributed near the mountains and/or hills, whereas the herbs and shrubs, dominated by Poaceae, grew on the plain and the aquatic herb *Typha* stood at the lakefront. Considering these factors, together with the rather low pollen concentration, the climate in this period was likely relatively cool and dry. This climate corresponds to the Last Glacial Maximum (LGM) (24-18 ka BP), when the global ice sheets reached their maximum integrated volume^58–61^. However, the pollen record of Guxu Lake (e.g., the lower PCA Factor 1 scores, the higher ratios of AP to NAP) exhibited a warming trend approximately 22 cal ka BP, which was anomalous within the regional context and was likely the result of local processes. Approximately 20 to 19 cal ka BP, a new warming period started and was coincident with continuous global warming at the end of the LGM^62–64^, which is also recorded in the Hulu Cave stalagmite^5^ and in the Greenland ice core^52^.

#### 18-11.7 cal ka BP, the Last Deglaciation

The Last Deglaciation (approximately 18-11.5 cal ka BP) is known for various high-frequency oscillations, such as the Bølling/Allerød warm periods^65,66^, the Heinrich event 1 (H1)^59,64,67,68^, the Oldest Dryas (OD)^65,69^ and the Younger Dryas (YD)^70–72^ cold phases, which are also reflected in the pollen assemblage from Guxu Lake. From 17.2 to 15.6 cal ka BP, the mixed evergreen-deciduous broadleaf and coniferous forest continued to shrink. However, herbaceous plants dominated by *Artemisia* and Poaceae expanded to a larger area and aquatic plants (mainly *Typha*) decreased sharply, suggesting that the climate in this period remained cold. The higher PCA Factor 1 scores, together with the lower ratios of AP/NAP associated with cooling and drying, are in strong agreement with the δ^18^O record of the stalagmites from the North Hemisphere^3,5,57^ and a Greenland ice core^52^, corresponding to the OD and/or H1 events, as is also recorded in the Greenland ice core and stalagmites^3,5,52,57^. During the period of 15.6-12.6 cal ka BP, the hills were covered by a mixed evergreen-deciduous broad leaved forest, and shrub and herbaceous plants were distributed in the plain and aquatic herbs dominated by *Typha*, together with fern spores, increased considerably, suggesting a relatively warmer and more humid climate, which is likely a reflection of the Bølling/Allerød warming oscillation. In addition, a notably cold and dry oscillation occurred during approximately 12.6-11.7 cal ka BP, as recorded in GDP Core 1. This oscillation is indicated by the reduction in the evergreen-deciduous broadleaf mixed forest, while herbaceous and shrub plants increased in conjunction with sharply decreased aquatic herbaceous and fern spores and decreased ratios of AP/NAP. This pattern likely correlates with the Younger Dryas event, which is also observed in the Chinese and Yemen stalagmites^3,5,53,57^, as well as in the Greenland ice core^52,65^.

#### 11.7-4.4 cal ka BP, the early and mid-Holocene

During this period, the mixed evergreen-deciduous broadleaf forest, represented by *Quercus* and *Castanopsis*, expanded to a larger area, with a small amount of *Ulmus*, *Juglans*, *Liquidambar*, *Betula*, *Alnus*, *Pinus*, and other taxa growing together in hilly areas. At the same time, terrestrial herbaceous and shrub plants (mainly Poaceae and *Artemisia*) continued to shrink, whereas aquatic herbaceous plants, dominated by *Typha*, increased considerably and spread throughout the lakefront. In addition, a small number of fern spores, represented by Polypodiaceae and *Ceratopteris*, grew under the forest. The abrupt decrease in the PCA Factor 1 scores, the increase in the ratios of AP/NAP, and the high total terrestrial and hydrophyte pollen concentrations during 11.7-7.9 cal ka BP suggest that the start of the Holocene was associated with warm and humid conditions. This hypothesis is consistent with the δ^18^O records from stalagmites in China and Oman^53–56^, as well as in the Greenland ice core^52,65,73^. Meanwhile, the charcoal record of the Guxu Lake presents some noteworthy issues. For example, the overall charcoal concentration in the Holocene is relatively high, especially that reached the peak in the Early Holocene, but there is no regional record of corresponding human activity. This is probably the result of the Holocene climate warming and vegetation development.

Then, 7.9-4.4 cal ka BP, which is known as the Holocene Optimum in the lower Yangtze River according to previous climatic and environmental studies of the middle Holocene^e.g.^^46,74–85^, a warmer and wetter climate was present, as indicated by pollen record. Both pollen and charcoal records indicate an increase in summer precipitation for the Taihu Lake Basin coincident with the Holocene summer insolation maxima^86^. However, the stalagmites in China recorded the wettest phase occurred during ca. 10.2-5.7 ka BP, which seems to indicate inconsistencies with the pollen records from the Guxu Lake (Fig. 6). This is consistent with the time-transgressive Holocene optimum mode^75^ though the duration of Holocene optimum has not been beyond debate^e.g.^ ^87^.

Multiple-plant remains analysis^88^ has been conducted on a Neolithic Yangjia site (ca. 6.3-5.9 cal ka BP) close to northeast of the Guxu Lake (Fig. 1, site 1). Pollen and phytolith records reveal the regional landscape at or around the site was a mosaic of *Quercus*-*Ulmus*-*Castanopsis*-Poaceae vegetation assemblage, and a general warm and humid conditions was present. This is consistent with that inferred from the nearby Guxu Lake. Besides, pollen analysis on other Neolithic cultural deposits in the Taihu Lake region reveals a relatively consistent vegetation landscape and environmental feature of the Majiabang Culture (7.0-5.8 ka BP).

However, a general weakening trend in the Asian monsoon during the Holocene caused by the change in insolation was punctuated by Asian Monsoon (AM) cooling events^6^, among which the 8.2 ka BP, 6.3 ka BP, 5.5 ka BP and 4.4-4.0 ka BP AM events were present in the pollen record of GDP Core 1. Among these events, the GDP Core 1 recorded suddenly reduced arboreal pollen percentage and the ratio of AP/NAP at around 8.2 ka BP (Fig. 6, Global chill), which is correlated with the “Global chill” cold event centered near 8.2 ka BP^89,90^. Cultural interruption during ca. 8.3-8.0 ka BP was witnessed at the Neolithic Kuahuqiao site^91^ (Fig. 1, site 10), which is likely to be affected by the cooling event. The end of this cooling event marked the beginning of the Holocene optimal in many areas.

The decrease of arboreal and total terrestrial pollen concentrations and the charcoal concentration at approximately 6.3 ka BP (Fig. 6, GDP 1) is also reflected in the δ^18^O record from the Dongge Cave stalagmite in eastern China^6^, which is correlated with an ice-rafted debris event^92^ and likely also attributed to preliminary anthropogenic perturbation from the Neolithic Majiabang Culture (7.0-5.8 ka BP) that occupied the (North) Taihu Lake Basin at approximately 6.5 ka BP. This is also present in pollen records of the profile of the Yangjia site^88^ (Fig. 1, site 1).

The cooling event occurred at approximately 5.5 ka BP (Fig. 6, Bond 4) and is correlated with the “Bond event 4” in the North Atlantic^92^, which is also documented in the δ^18^O record of stalagmites in eastern China and Oman^6,55,56^. This event presumably accelerated cultural development in Neolithic China, especially the Liangzhu Culture (5.5-4.3 ka BP), and even indirectly caused the emergence of civilizations in Egypt and Mesopotamia^93^. In addition, the suddenly changing pollen record and abrupt reduced charcoal concentration at approximately 5.0 ka BP (Fig. 6, GDP 2) was probably attributed to expanding and intensified human activities during the Liangzhu Culture according to archaeological records.

#### After 4.4 cal ka BP, the late Holocene

The former phase of this stage (4.4-1.9 cal ka BP) presented a shrinking of the subtropical broadleaf evergreen-deciduous mixed forest with an expansion of herbaceous plants, especially Poaceae, suggesting relatively cool and dry conditions, which correspond to a cold event approximately 4.2 ka BP in South China^94^ and Neoglacial episodes^61,95^. Climatic change at 4.4-4.0 ka BP, known as “Holocene Event 3” or “Bond events 3” (Fig. 6, Bond 3)^92,96,97^, is presumed to have played a very important role in the shift of Neolithic cultures in China^98,99^. In particular, the collapse of the Liangzhu Culture in the Taihu Lake Basin is considered to have resulted directly from this cooling event^100,101^. This hypothesis is consistent with the pollen record from Guxu Lake and the δ^18^O records of stalagmites from Dongge Cave and Oman^6,55,56^.

In addition, during the late phase (1.9-0 cal ka BP), the forest was dominated by broadleaved evergreen *Castanopsis* and *Quercus* growing on the surrounding hills, with Poaceae and *Artemisia* on the plain and a small population of aquatic herbaceous *Typha* on the lake shore. Nevertheless, the notable change in the pollen assemblage and concentration reflects a more intensive human influence on local vegetation and the environment, though it might be correlate with the Little Ice Age^102,103^ during its early period.

### Occurrence and development of rice agriculture

Though pollen of Poaceae comprises a small portion overall, it is can still be used as a reference when it is the dominant terrestrial herb, especially combined with so-called *Oryza*-type and wild grass. In the Lower Yangtze River, Poaceae pollen with a grain diameter is greater than 40 μm and an aperture diameter wider than 4.0 μm is generally identified as *Oryza*-type Poaceae^32,104–106^, which will benefit our discussion on rice collection, utilization and even cultivation. In general, proxy data of rice pollen extracted from the Guxu Lake sediments present changes over time (Fig. 6G,H). Prior to the Holocene (before 11.7 cal ka BP), the presence of rice pollen in GDP Core 1 together with a certain amount of rice pollen and wild rice phytoliths present in the parent soil of the nearby Yangjia site^88^, may suggest that wild rice was once distributed in the surroundings of Guxu Lake before human occupation. In the early Holocene (11.7-7.9 cal ka BP), rice pollen was distributed with relatively lower percentage and notably higher concentration, which was most likely due to the warm and humid climate that accelerated the natural distribution and growth of wild rice. During the mid-Holocene (7.9-4.4 cal ka BP), the decreased rice pollen percentage and concentration may correlate with human occupation and rice agriculture that occurred and developed during this period. Moreover, a relatively high concentration of charcoal, approximately 1.868 cm^2^ per gram dry sample on average, might also reflect perturbation by humans. Correspondingly, rice remains unearthed from the nearby Yangjia site^88^, including charred rice grains, domesticated rice spikelets, rice phytoliths from rice husks and leaves, as well as rice pollen, illustrated a scenery of rice farming. In addition, a large number of previously excavated Neolithic archaeological sites and previous studies on rice agriculture in this area^e.g.^ ^30,31,107–113^ were dated to have begun and/or developed in the early and middle part of the Holocene (ca. 7.0-4.0 ka BP), which is in agreement with our results. In the late Holocene (after 4.4 cal ka BP), the significantly increased rice pollen percentage and concentration together with high concentration of charcoal is a clear signal of intensified anthropogenic disturbance that corresponds to widespread intensified farming activities and the control of local fire events in historical periods as well as relatively stable environmental conditions.

**Figure 7:**
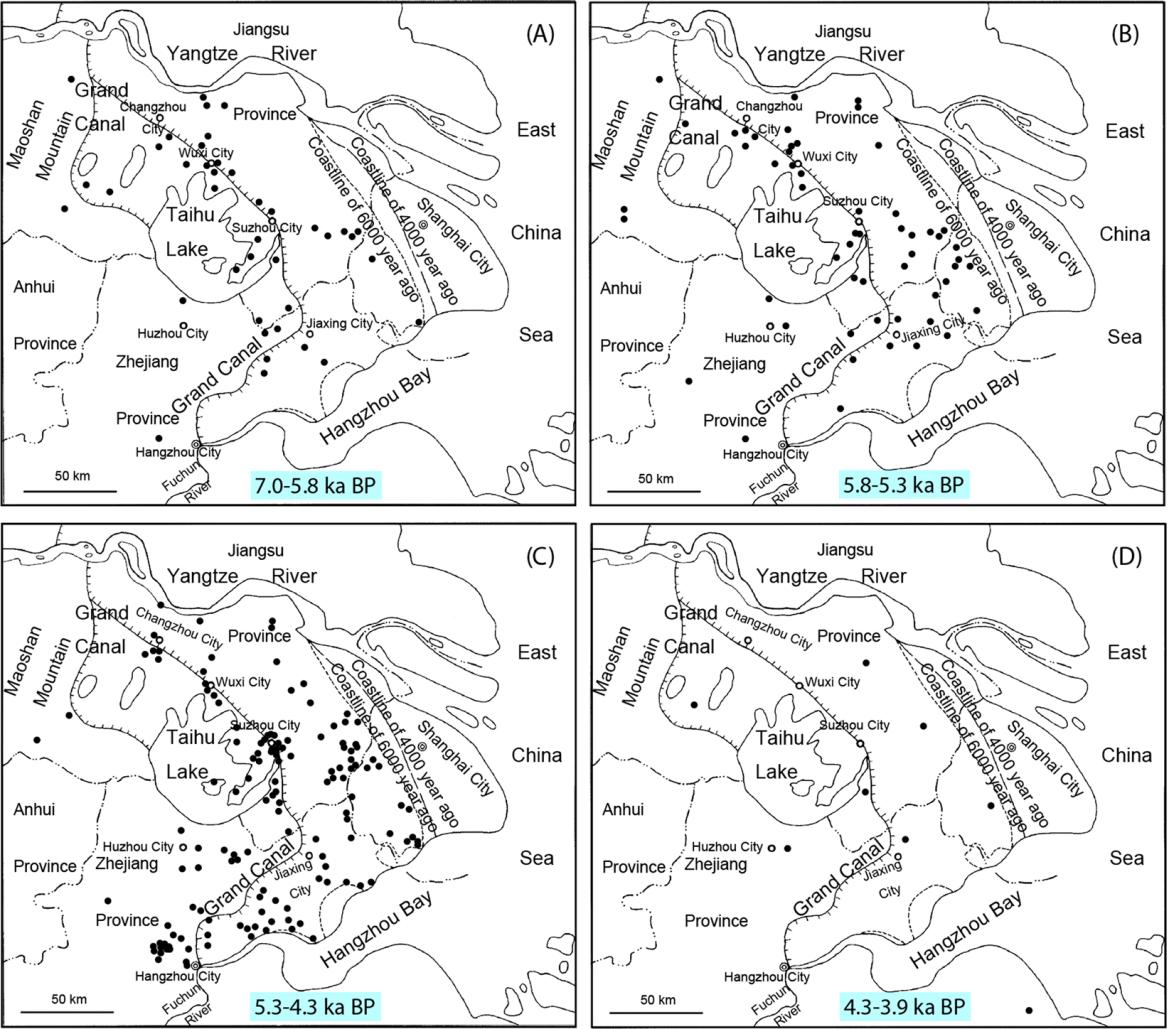
The distribution of Neolithic archaeological sites in the Taihu Lake area plotted for the main cultural periods (modified from IACASS^117^). (**A**) 40 sites of the Majiabang Culture period (7.0-5.8 ka BP); (**B**) 55 sites of the Songze Culture period (5.8-5.3 ka BP); (**C**) 248 sites of the Liangzhu Culture period (5.3-4.3 ka BP); (**D**) 8 sites of the Guangfulin-Qianshanyang Culture period (4.3-3.9 ka BP).

The presence and level of rice agriculture in the Taihu Lake area, as an example of typical human activities, could be reflected to some extent by the number and size of Neolithic human occupations. Based on Neolithic archaeological sites in this area^114–117^ and the latest archaeological excavation reports, plots were drawn to demonstrate changes in the quantity and distribution density of 331 known archaeological sites in different periods. Generally, Neolithic archaeological cultures in this area can be divided into three main periods, namely, the Majiabang Culture (7.0-5.8 ka BP), Songze Culture (5.8-5.3 ka BP), and Liangzhu Culture (5.3-4.3 ka BP). The number and density of sites increased significantly from the Majiabang Culture (40) to the Liangzhu Culture (243), then decreased sharply to 8 for the Guangfulin-Qianshanyang Culture (4.3-3.9 ka BP) (Fig. 7), illustrating the intensified human activities in the Taihu Lake area during the most of the Neolithic period (ca. 7.0-4.2 ka BP), which is in good agreement with the Holocene Optimum (Fig. 6).

In this case, human settled locally when the Guxu Lake was relatively stable (ca. 8.0-6.5 cal ka BP) as reflected by the hydrophyte concentration (Fig. 6E). Meanwhile, the first occurrence of human activities since the Majiabang Culture was recorded in the vegetation landscape, such as changing rice pollen and charcoal concentration illustrated in the GDP Core 1 (Fig. 6, GDP 1). In the second stage, the Songze Culture developed slowly, which might be related to the “Bond 4” cold event around 5.5 ka BP. Then, the rapid development of Liangzhu Culture witnessed a fine social structure system consisted of dam, city, noble cemetery, exquisite jade, and developed paddy field agriculture in generally stable warm and humid conditions during 5.3-4.3 ka BP as recorded in the Guxu Lake sediments. Especially the abrupt reduction of pollen and charcoal concentration around 5.0 ka BP, is probably a reflection of expanding and intensified human interference with natural vegetation. However, a considerable shift occurred at the end of the Neolithic, i.e., the collapse of the Liangzhu Culture. This is coincident with the abrupt cooling event at 4.4-4.0 ka BP, which might exacerbate change within prehistoric society in the Taihu Lake region.

Generally, ‘slash-and-burn’ cultivation appeared beginning ca. 7.0 ka BP (early Majiabang Culture) and rice agricultural activities expanded in the Taihu Lake area during the Neolithic. Expanding and intensified anthropogenic activities affected the distribution of arboreal plants, and probably resulted in decreased charcoal concentration. Wild rice was supposed to be distributed in this area before Neolithic humans occupied the area, and it may have been collected as supplementary food together with cultivated rice as the main plant food resources, especially during the Liangzhu Culture period.

## Conclusion

Changes in vegetation on the East Asian continent were considered to controlled primarily by the East Asian monsoon, including the East Asian summer and winter monsoons^118^. In the Northern Hemisphere, orbital forcing of insolation increased in the summer and decreased in the winter during the early- to mid-Holocene, causing generally warmer summers and colder winters compared with the present^119^. Variations in pollen and charcoal records, in conjunction with changes in the sedimentary lithology from a core at Guxu Lake, yielded evidence of several shifts in vegetation belts as well as rice farming, allowing for a reconstruction of the vegetation of the last 23 cal ka BP and a recovery of Neolithic rice agriculture in this area. During the period of ca. 23.0-15.6 cal ka BP, forest-savanna mosaics developed, reflecting a stronger winter monsoon. In correspondence with the strengthening of the Asian summer monsoon, subtropical forests quickly developed from approximately 15.6 cal ka BP to 4.4 cal ka BP, as reflected in the δ^18^O records from the Sanbao Cave, Hulu Cave and Dongge Cave stalagmites in central and eastern China^3,5,6,53,54^, Yemen^57^ and Oman^55,56^, as well as from Greenland ice cores^52,65,73^.

Our main inferences on vegetation evolution and human dynamics are as follows:

1. The Late Pleistocene was characterized by Poaceae-*Quercus*-*Castanopsis* taxa, the climate in this period was relatively cool and dry. Additionally, the major post-glacial climatic events are clearly identified in the sequence, including the Late Glacial series of the Heinrich event 1, Bölling/Alleröd, and Younger Dryas periods.
2. During the Early and Middle Holocene, the regional landscape was a mosaic of forest, grass and (rice) croplands rich in *Quercus*-*Castanopsis*-Poaceae-*Artemisia*-*Typha* vegetation, a warmer and more humid climate was present in this period, and the Holocene Optimum appeared during 7.9-4.4 cal ka BP in this area.
3. The vegetation landscape (mainly *Castanopsis*-*Quercus*-Poaceae) since the Late Holocene (ca. 4.4 cal ka BP) has been comparatively stable and is close to that of today.
4. Wild rice may have been distributed in this area even before the Holocene, and rice agriculture occurred and developed during the Holocene Optimum period. The occurrence and expanding of anthropogenic activities affected the vegetation landscape.
5. After 4.4 cal ka BP, rice farming spread over this area and a more intensified human influence affected the local environment.

## Methods

### Sample collection and description

According to preliminary surveys and drilling data, we selected the relatively thick sedimentary location (31°30′47.8″N, 120°07′13.4″E, 14.6 m a.s.l.) of Guxu Lake to carry out the Guxu Drilling Project (GDP) in March 2013 and obtained an 18.40-m-long sediment core. Specific lithological features are described as in Table 3.

**Table 3:** Sedimentary features of GDP Core 1 of Guxu Lake.

Depth (cm)	Sedimentary features
0–20	Gray brown coarse silt with plenty of plant rootlets.
20–45	Gray silt with dark spots.
45–55	Light yellow brown silt with a small amount of plant rootlets.
55–63	Gray silt with a small amount of plant rootlets.
63–78	Yellow brown silt with a small amount of plant rootlets.
78–87	Gray brown silt with a small amount plant of rootlets.
87–105	Gray silt with plenty of yellow rust spots. The lower part (98–105 cm) is characterized by dark gray silt and less rust spots.
105–145	Bluish gray silt with yellow rust spots. The lower part (125–143 cm) is characterized by more rust spots while the bottom (143–145 cm) is dark gray silt and no rust spots.
145–165	Yellow brown silt.
165–230	Black grey silt with plenty of plant rootlets, seeds and charcoals.
230–288	Gray silt with plenty of plant rootlets, seeds and charcoals.
288–397	Bluish gray silt with a small amount of plant rootlets.
397–405	Gray silt with a very small amount of plant rootlets.
405–432	Dark gray fine silt with plenty of plant rootlets and charcoals.
432–517	Gray silt with plenty of plant rootlets and charcoals.
517–553	Bluish gray silt with plenty of plant rootlets and charcoals.
553–633	Gray silt.
633–673	Bluish gray silt with a small amount of yellow rust spots.
673–689	Grayish green silt with plenty of yellow rust spots.
689–725	Gray silt.
725–759	Bluish gray silt. The upper part (725–749 cm) is characterized by more fine bluish gray silt.
759–826	Gray brown silt with a very small amount of plant rootlets.
826–870	Yellow brown silt with a very small amount of plant rootlets. The upper part (826–846 cm) is characterized by bluish gray patches.
870–916	Gray brown silt with a small amount of plant rootlets.
916–940	Gray silt with a small amount of plant rootlets.
940–950	Gray brown silt with a very small amount of plant rootlets.
950–963	Bluish gray silt with a very small amount of plant rootlets.
963–1090	Gray brown silt with a small amount of plant rootlets and charcoals.
1090–1175	Bluish gray silt.
1175–1226	Gray silt with a small amount of plant rootlets.
1226–1406	Gray brown silt. The lower part (1369–1406 cm) is characterized by dark brown patches.
1406–1446	Black grey silt with plenty of plant rootlets.
1446–1481	Bluish gray silt with a small amount of plant rootlets.
1481–1526	Dark bluish gray fine silt with a very small amount of plant rootlets.
1526–1606	Bluish gray silt. The upper part (1526–1566 cm) is characterized by much yellow rust spots while the lower part (1566–1606 cm) doped with less light yellow rust spots.
1606–1626	Bluish gray silt. The upper part (1606–1609 cm) is more dark.
1626–1676	Gray brown silt.
1676–1840	Yellow brown fine silt. The lower part (1810–1840 cm) is more dark.

Generally, the sediments between 17 m and 2 m of the core at Guxu Lake are characterized by light or dark gray silt intercalated with bluish gray silt and gray brown silt interbeds. We divided the core into samples for pollen, phytolith and other analyses per 2 cm segments and chose the upper 10 m with intervals of 10 cm for pollen analysis (101 samples in total).

### Dating

Organic sedimentary samples were dated with an accelerator mass spectrometer (AMS) using ^14^C in the Beta Analytic Radiocarbon Dating Laboratory (Miami, Florida, USA) and Radiocarbon Dating Laboratory at Peking University (Beijing, China) respectively, then calibrated using IntCal13^120^ and OxCal v4.3.2^121^ to convert the radiocarbon ages to calendar years.

### Pollen and charcoal analysis

A palynological analysis was carried out on the basis of procedures suggested or applied by Moore, *et al*.^122^ and Horrocks^123^. Samples of 5 g dried powder were processed with HCl (37%, 50 ml), KOH (10%, 50 ml), KI/IH (2.0 g/ml in density, 5 ml), acetic acid (50 ml) and an acetolysis mixture (1 ml concentrated H_2_SO_4_, 9 ml acetic anhydride). A tablet of *Lycopodium* marker (20,848 grains) was added to each sample. The pollen samples were spread uniformly on glass slides, and at least 500 pollen grains, excluding aquatic pollen and spores, were identified and counted using a Nikon Eclipse LV100POL microscope. Identification was aided by the use of reference materials collected by the Key Laboratory of Vertebrate Evolution and Human Origins of Chinese Academy of Sciences, Institute of Vertebrate Paleontology and Paleoanthropology, Chinese Academy of Sciences and published keys^124^. Charcoal analyses were carried out as part of the routine pollen analysis^125^. Charcoal content was estimated using the point count method described by Clark^126^ by counting the proportion of 5,500 points of charcoal that were touching an 11-point ocular scale. To reduce potential error from small dark particles that are difficult to assign as charcoal, only points falling on particles greater than 5 μm were counted. Diagrams were constructed using Tilia^127,128^ and zoned according to variations in pollen percentages and concentrations using CONISS^129^.

### Multivariate analysis

To obtain information regarding similar pollen composition among different pollen zones, as well as to determine which pollen taxa exhibited similar reactions in the diagram of pollen percentage, a detrended correspondence analysis (DCA) and principal component analysis (PCA) were conducted based on the square-root-transformed pollen percentage data (except aquatics) using CANOCO 5 software^130^ for ordination and plotting a “species-sample bi-plot.” The DCA showed that the pollen percentage data set has a mainly linear structure, resulting in the use of a linear-based PCA^131^. In the PCA, the data were “species-centered.”
